# Shared Air: A Renewed Focus on Ventilation for the Prevention of Tuberculosis Transmission

**DOI:** 10.1371/journal.pone.0096334

**Published:** 2014-05-07

**Authors:** Eugene T. Richardson, Carl D. Morrow, Darryl B. Kalil, Linda-Gail Bekker, Robin Wood

**Affiliations:** 1 Division of Infectious Diseases and Geographic Medicine, Stanford University School of Medicine, Stanford, California, United States of America; 2 Department of Anthropology, Stanford University, Stanford, California, United States of America; 3 Desmond Tutu HIV Centre, Institute of Infectious Diseases and Molecular Medicine, University of Cape Town, Cape Town, Republic of South Africa; University of Calgary & ProvLab Alberta, Canada

## Abstract

**Background:**

Despite an improvement in the overall TB cure rate from 40–74% between 1995 and 2011, TB incidence in South Africa continues to increase. The epidemic is notably disquieting in schools because the vulnerable population is compelled to be present. Older learners (age 15–19) are at particular risk given a smear-positive rate of 427 per 100,000 per year and the significant amount of time they spend indoors. High schools are therefore important locations for potential TB infection and thus prevention efforts.

**Methods and Findings:**

Using portable carbon dioxide monitors, we measured CO_2_ in classrooms under non-steady state conditions. The threshold for tuberculosis transmission was estimated using a carbon dioxide-based risk equation. We determined a critical rebreathed fraction of carbon dioxide (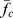) of 1·6%, which correlates with an indoor CO_2_ concentration of 1000 ppm. These values correspond with a ventilation rate of 8·6 l/s per person or 12 air exchanges per hour (ACH) for standard classrooms of 180 m^3^.

**Conclusions:**

Given the high smear positive rate of high-school adolescents in South Africa, the proposal to achieve CO_2_ levels of 1000ppm through natural ventilation (in the amount 12 ACH) will not only help achieve WHO guidelines for providing children with healthy indoor environments, it will also provide a low-cost intervention for helping control the TB epidemic in areas of high prevalence.

## Introduction

**Figure 1:**
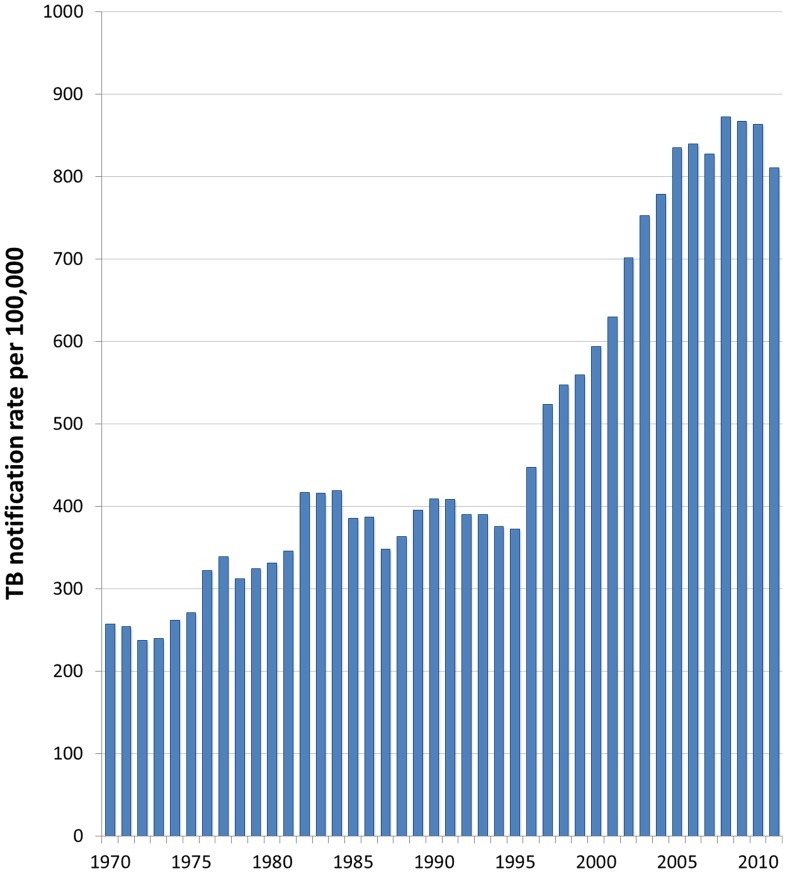
TB Notification Rates for Cape Town, 1970–2011 [5].

Once the infectious origin of tuberculosis was discovered by Koch in 1882, schemes for improving indoor ventilation spread across the industrialized world [1]; such plans were *associated* with dramatic declines in TB incidence [2]. South Africa, however, currently has tuberculosis notification rates similar to those reported in 19^th^ century industrializing nations [3]. Despite successful TB case identification and treatment programs, the country had an incidence greater than 1,000 per 100,000 people in 2012 [4]. This high incidence is not fully explained by HIV, as it was increasing prior to the start of the South African HIV epidemic in 1990 (see Figure 1) [5].

Many South African communities have yet to benefit from the campaign for healthy indoor environments found in high-income countries. And while the post-apartheid government in South Africa has done an excellent job formalizing 206 of the 2,700 informal settlements countrywide [6], a substantial number of South Africans still live in the equivalent of the 19^th^ century industrial tenement housing described by Marx and Engels [7]. Thus, a plausible explanation for the high prevalence is the continued existence of crowded, poorly ventilated indoor environments.

In 2012, young people aged 15–24 represented 17·2% of all smear-positive cases in South Africa. Despite an improvement in the overall TB cure rate from 40–74% between 1995 and 2011, TB incidence continues to increase [4]. The epidemic is notably disquieting in schools because—not unlike prisons [8]—the vulnerable population is compelled to be present. Older learners (age 15–19) are at particular risk given a smear-positive rate of 427 per 100,000 [4], [9] of their peers and the significant amount of time they spend indoors at school [10]. High schools are therefore important locations for potential TB infection and thus appropriate targets for prevention efforts.

The force of infection for tuberculosis in Cape Town has been calculated to be at least 6% per annum in people aged 15–19 [11]. The number of individuals infected by each case (effective contact number) is determined by the ratio of the force of infection (6%) and the prevalence of infectious TB cases (427/100,000). For high school students in Cape Town, the calculated effective contact number per case is 14. Effective TB control is achieved when the effective contact number is lower than the number of individuals who can be expected to develop infectious pulmonary TB over a lifetime. Thus, the effective contact number per case must be reduced to <14 to decrease the current TB burden.

Re-circulated indoor air has long been recognized as a mechanism for infectious disease transmission [12]. By measuring carbon dioxide levels in classrooms, one can estimate probabilities of TB transmission using the equation developed by Rudnick and Milton (see Methods):

Exhaled breath is the vehicle for release of airborne infectious particles. Exhaled breath contains almost 40,000 ppm of CO_2_ compared with approximately 350 ppm [currently 400 ppm due to anthropogenic global change] in outdoor air [13].

Their calculation incorporates CO_2_ as a surrogate for exhaled breath, thereby addressing two key limitations of the Wells-Riley equation [14] on which it was based. First, the Rudnick-Milton adaptation does not require the assumption of steady-state conditions. Second, it assumes the loss of infectious particles to settling, filtration, and loss of viability is negligible compared to that removed by ventilation [13].

Recent studies utilize the Wells-Riley equation to determine ventilation recommendations for TB prevention but are based on the assumption of steady state conditions [15], [16]. We believe this is the first publication to date to use the Rudnick-Milton equation to determine ventilation recommendations for the transmission of tuberculosis under non-steady state conditions in schools.

## Methods

### Ethics Statement

Ethics approval for this study was obtained from the Human Research Ethics Committee at the Faculty of Health Sciences, University of Cape Town. For minors enrolled in the study, we obtained written informed consent from a parent or guardian. This consent procedure was approved by the Human Research Ethics Committee.

### Equations Used for Estimation of Transmission Risk

**Figure 2:**
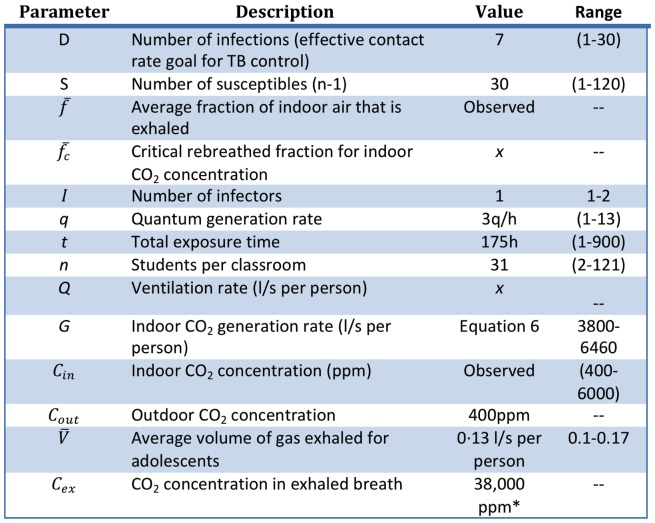
Parameter definitions and values used in computing CO_2_ threshold and ventilation rates [18]–[22], [24], [38]–[40]. *ppm (parts per million) = mg/l



The threshold for tuberculosis transmission was estimated using the carbon dioxide-based risk equation developed by Rudnick and Milton (refer to Figure 2 for descriptions of parameters) [13].

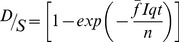
(1)The conversion between indoor CO_2_ concentration and ventilation rate is expressed by [17]

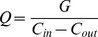
(2)


The critical rebreathed fraction (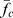) represents the fraction of ambient CO_2_ under which a reduction in TB transmission would be expected to occur. Substituting (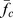) for the indoor CO_2_ concentration yields
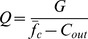
(3)


The indoor CO_2_ generation rate is expressed by
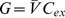
(4)


The ventilation rate can be converted to air changes per hour (ACH) by
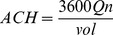
(5)


### Data Collection Procedures

Using portable carbon dioxide detection devices (EasyView 80 CO_2_ analyzer, Extech Instruments, Waltham, MA) and custom monitors (based on COZIR Ambient sensors, Gas Sensing Solutions Ltd., Glasgow, UK), we monitored CO_2_ in non-mechanically ventilated classrooms in a high TB burden community *under varying natural conditions*. The accuracy of CO_2_ measurements taken by the sensor is reported by the factory to be ±50 ppm or 3% of each reading (www.cozir.com). To verify that devices were not affected by the wearer's respiration, we conducted an experiment measuring CO_2_ levels in an unventilated space with one individual using CO_2_ devices, placed on areas of the body where our study participants wore their devices. From these trials, we were not able to find differences in data from monitors hung from a neck lanyard or monitors worn in waist pockets—the two locations where subjects were instructed to keep the devices on their person.

Our sample consisted of 64 students carrying individual monitors over 91 school days throughout an entire school year (for a total of 509 hours of school time). The monitors provide a CO_2_ measurement in parts per million (ppm) every 60 seconds as well as GPS locations. The average number of students per class (*n*) was 31. We estimated *q* using the value obtained in previous studies [18]–[22] combined with the logic that—since the molecular epidemiology of TB in Cape Town militates against the presence of super-spreaders [23]—*q* would not be at the high levels found in some hospital outbreaks. We also assumed that infectious cases would overlap with the same individuals for up to 175 hours of class time (i.e., 35 school days [24] at 5 indoor hours per day) before diagnosis. We then solved for the critical rebreathed fraction (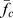).

### Solving for the Critical Rebreathed Fraction

We calculated that the effective contact number per case must be reduced to <14 to reduce the current TB burden (see introduction). Since 50% of transmission for 15–19 year-olds occurs in schools [25], the effective contact number for schools is 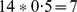 (i.e., the portion of contacts in a TB case's total social-network that he/she infects in school). Setting D = 7 for 30 susceptibles (Equation 1) and solving for the critical rebreathed fraction (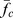) yields
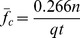
(6)


## Results

By substituting the values in Figure 2, we obtain a critical rebreathed fraction (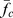) of 1·6%, which correlates with an indoor CO_2_ concentration of 1000 ppm. Entering this value into equation 3 yields a ventilation rate of 8·6 l/s per person. Note that classes are more likely to approach steady-state conditions when highly ventilated.

Using equation 5, the value 8·6 l/s per person (with an average class size of 31 students and class volume of 180,000 liters or 180 m^3^) [26] converts to between 5 and 6 air changes per hour (ACH), which is around half the level of ventilation recommended for health care settings [27], [28].

**Figure 3:**
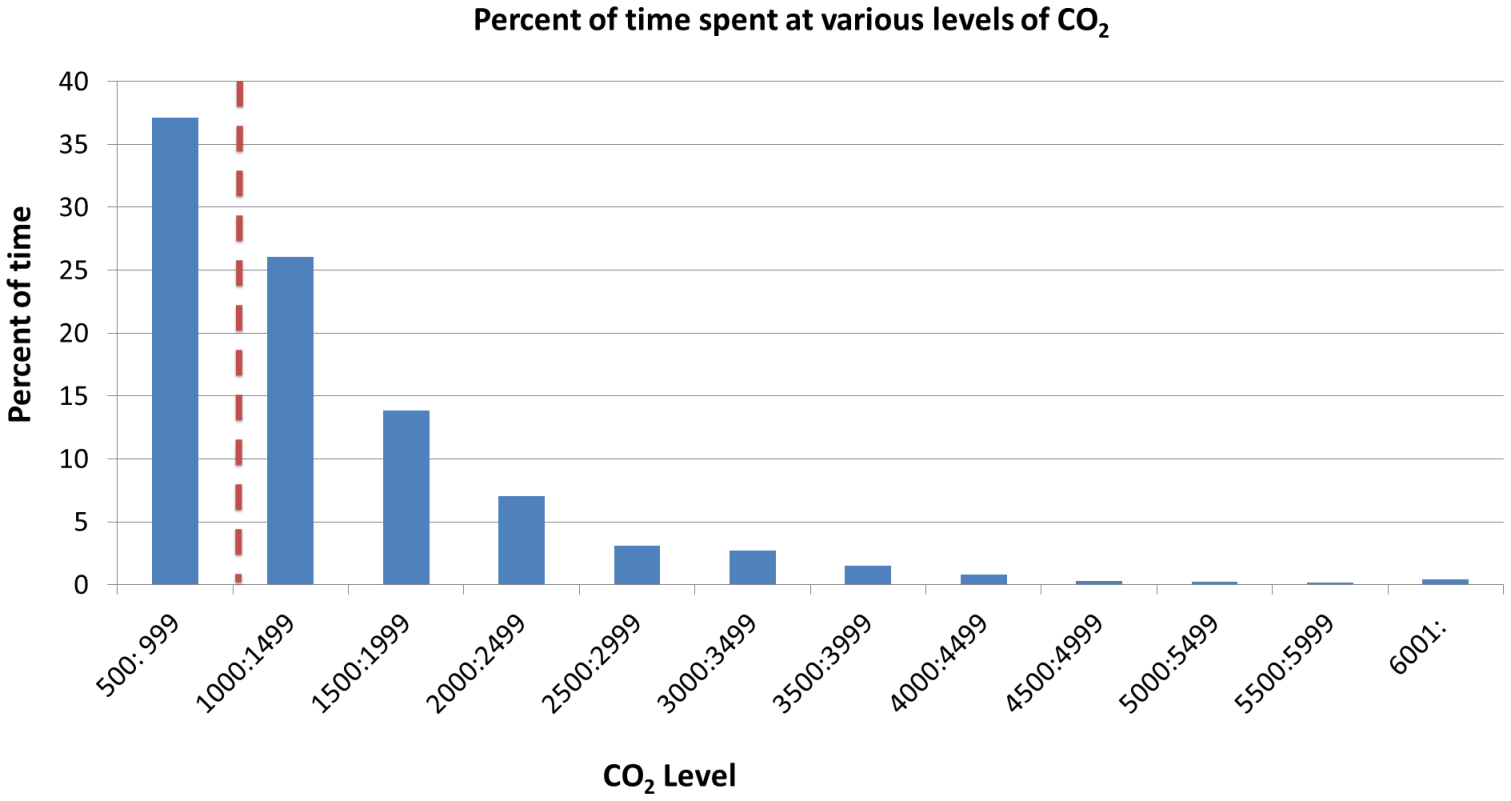
The percent of time spent at various levels of total CO_2_ (in ppm). CO_2_ levels are shown in ranges of 500ppm. The dotted red line represents calculated threshold for reducing TB transmission. Sample: 64 students over 91 school days (509 class hours).

To explore the ramifications of our recommendations in the local context, we conducted CO_2_ analyses in classrooms in an area of high TB prevalence in Cape Town, South Africa. Our findings demonstrate that students spend 60·2% of their time above our recommended threshold (Figure 3).

**Figure 4:**
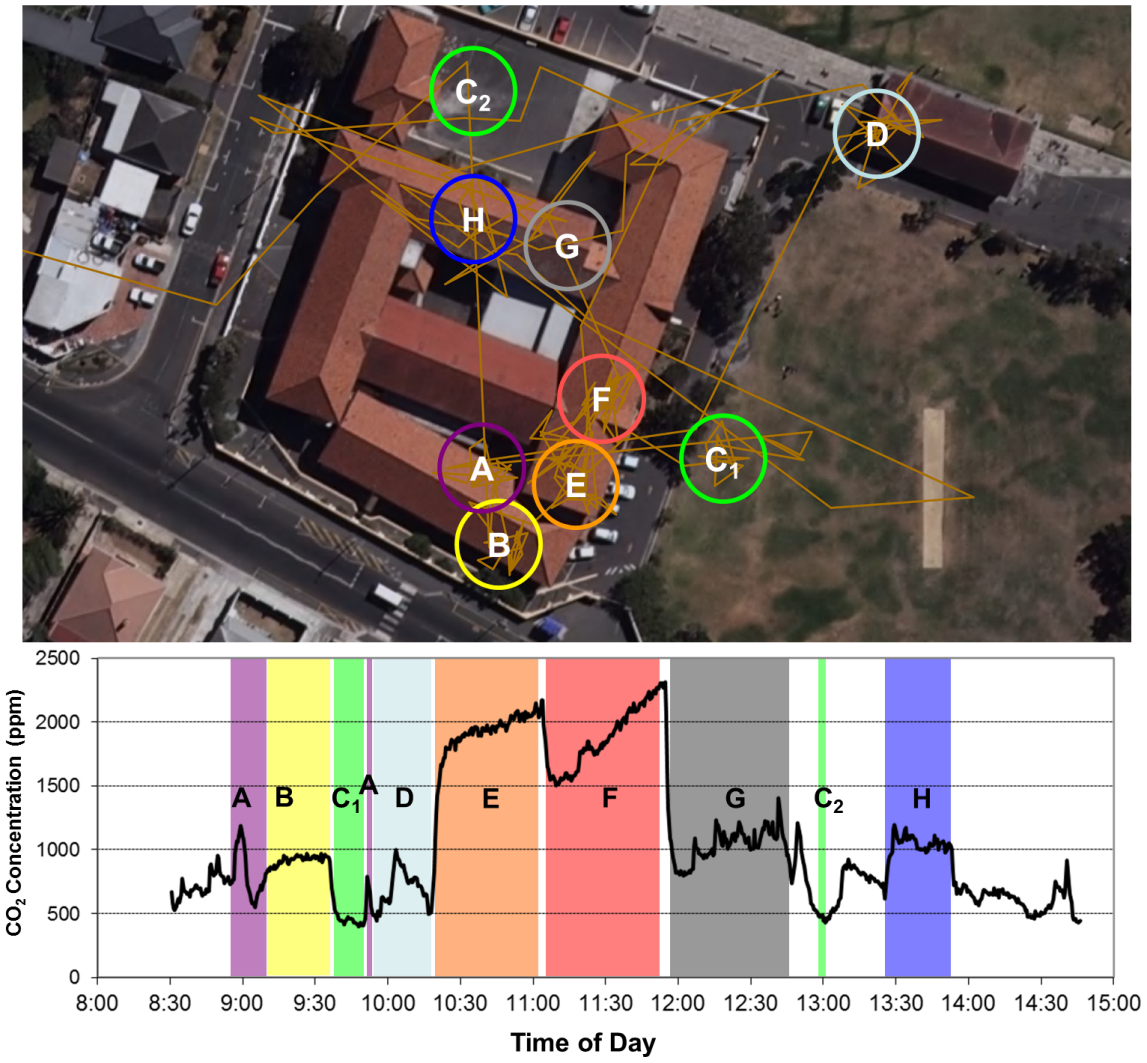
Sample student day with measured CO_2_ concentrations and GPS locations (A–H).

A sample student day with CO_2_ measurements and GPS locations is presented in Figure 4. Visits to different classrooms and outside locations throughout the day are clearly identifiable. In addition, the CO_2_ environment encountered is seen to be highly variable. Classrooms B, H and G achieve steady state conditions quite quickly. In spite of very high levels of CO_2_, classrooms E and F do not achieve steady-state conditions (these are the only classes visited without windows on more than one side of the room).

## Discussion

In this article, we have presented a statistical buttressing of recommendations made over a century ago. It seems that—in an era of effective treatment—current TB prevention programs have become complacent in promoting the prevention benefits of ventilation. There is growing consensus that biomedical solutions will be insufficient to tackle the TB epidemic in low and middle income countries [29]. Thus, a renewed focus on environmental interventions is called for. Given the high smear positive rate of high-school adolescents in Cape Town informal settlements, the proposal to achieve CO_2_ levels of 1000ppm through natural ventilation (in the amount 12 ACH for a standard classroom of 180 m^3^) will not only help achieve WHO guidelines for providing children with healthy indoor environments, it will also help curb the TB epidemic the ‘old-fashioned way’ (i.e., through improvements in air hygiene).

**Figure 5:**
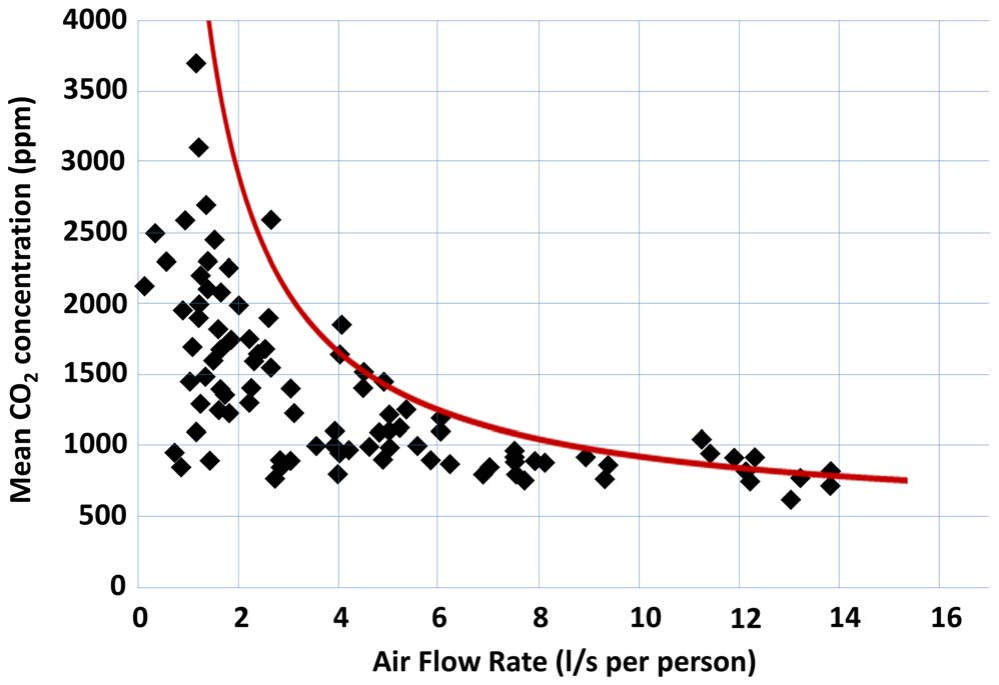
Recommended ventilation rates in classrooms by country [41]–[45]. * Denotes calculated value.

**Figure 6:**
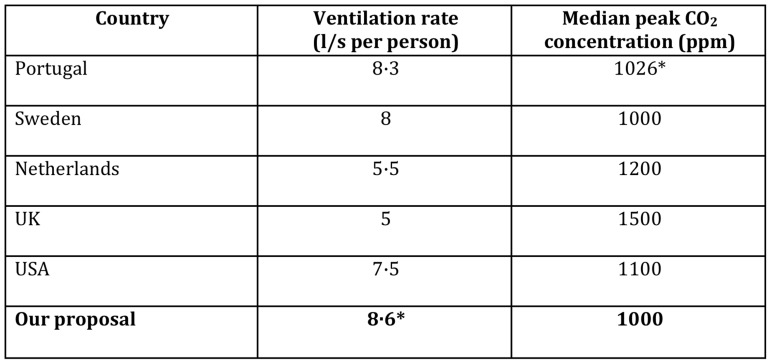
Correlation between the mean indoor CO_2_ concentrations in naturally ventilated classrooms and the air flow (l/s per person). Red line represents relationship between air flow and mean CO_2_ concentration at steady state (defined by Equation 2). Adapted from M. Santamouris *et al*. (2008) [33].

The cutoff of 1000 ppm falls in line with regulations in other industrialized nations (Figure 5). It should be recognized that the benefits of increased ventilation are not limited to prevention of TB transmission: decreased respiratory illness, fewer school absences, and improved cognitive function have been demonstrated in the literature [30]–[32]. South Africa is not alone in failing to adequately ventilate schools (although the consequences may be greater due to the high TB incidence). The majority of European classrooms in a meta-analysis by Santamouris *et al*., were found to be inadequately ventilated (see Figure 6) [33]. Furthermore, the occurrence of TB outbreaks in schools in middle- to high-income countries suggests a renewed focus on ventilation recommendations is apposite across a broad range of settings [34]–[36].

Given historic precedent, pharmacologic intervention on its own is unlikely to curtail the TB epidemic. This work would suggest that the addition of a key structural intervention such as improved school ventilation might enhance our efforts to effect TB control. Indeed, given the association of formal recommendations by Commissions on Ventilation with decreased TB notifications in industrialized nations [37], it may be prudent to revive such commissions in areas of high TB prevalence.

### Limitations

1. We did not measure CO_2_ concentrations in a given classroom with multiple devices simultaneously and thus cannot provide an error analysis for our measurements; however, the devices were factory calibrated, and we periodically compared the performance of monitors (in our offices) and found them to be consistently measuring within 10% of each other.

2. We did not record temperature so do not have temperature associations with the varying CO_2_ levels found in classrooms; however, we did conduct the study over a full year and thus control for the effect of a whole year's weather on classroom ventilation (i.e., number of windows open).

3. As shown in Figure 2, there is significant variation in the parameters used to determine the TB risk threshold; however, we feel that the use of empirically derived numbers for a high-incidence community coupled with conservative choices for the theoretical parameters allows us to calculate a threshold for tuberculosis transmission that is evidence-based as well as practically meaningful.
