# Correction: Shared air: A renewed focus on ventilation for the prevention of tuberculosis transmission

**DOI:** 10.1371/journal.pone.0106216

**Published:** 2014-08-19

**Authors:** The PLOS ONE Staff

Samuel Ginsberg is missing from the original author list. He should be listed as the fourth author, and his affiliation is 3: Desmond Tutu HIV Centre, Institute of Infectious Diseases and Molecular Medicine, University of Cape Town, Cape Town, Republic of South Africa. The contributions of this author are as follows: Contributed reagents/materials/analysis tools.

The correct citation is: Richardson ET, Morrow CD, Kalil DB, Ginsberg S, Bekker L-G (2014) Shared Air: A Renewed Focus on Ventilation for the Prevention of Tuberculosis Transmission. PLoS ONE 9(5): e96334. doi:10.1371/journal.pone.0096334

The images for Figure 5 and Figure 6 are incorrectly switched. The image that appears as Figure 5 should be Figure 6, and the image that appears as Figure 6 should be Figure 5. The figure legends appear in the correct order. The correct Figure 5 and Figure 6 with legends, can be viewed here.

**Figure 5:**
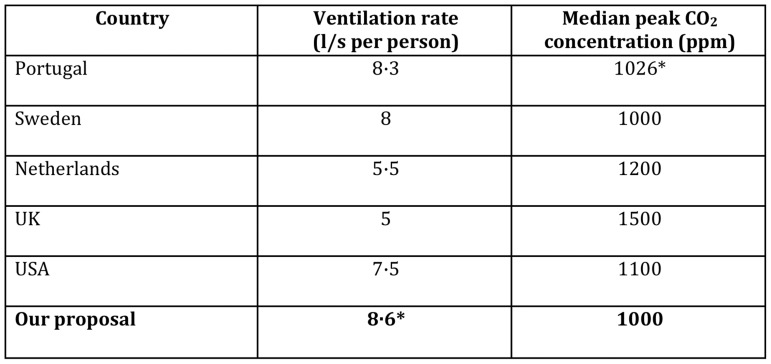
Recommended ventilation rates in classrooms by country [40–44]. * Denotes calculated value.

**Figure 6:**
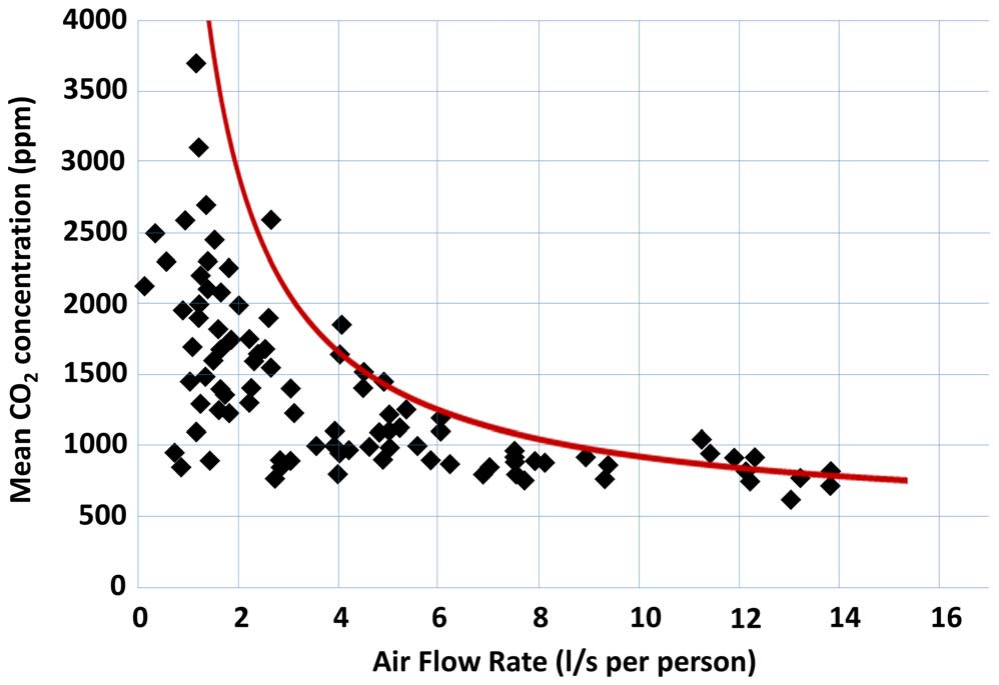
Correlation between the mean indoor CO_2_ concentrations in naturally ventilated classrooms and the air flow (l/s per person). Red line represents relationship between air flow and mean CO_2_ concentration at steady state (defined by Equation 2). Adapted from M. Santamouris *et al*. (2008) [33].
