# Correction: Correction: Shared air: A renewed focus on ventilation for the prevention of tuberculosis transmission

**DOI:** 10.1371/journal.pone.0110017

**Published:** 2014-09-30

**Authors:** The PLOS ONE Staff

There is an error in the Correction published on August 19, 2014. The affiliation for Samuel Ginsberg is incorrect. Samuel Ginsberg is not affiliated with #3, please refer to his correct affiliation here: Department of Electrical Engineering, University of Cape Town.
